# Novel Discovery of the Somatostatin Receptor (SSTR2) in Pleomorphic Adenomas via Immunohistochemical Analysis of Tumors of the Salivary Glands

**DOI:** 10.3390/cancers15153917

**Published:** 2023-08-01

**Authors:** Felix Johnson, Benedikt Hofauer, Markus Wirth, Barbara Wollenberg, Fabian Stögbauer, Susan Notohamiprodjo, Bernhard Haller, Robin Reschke, Andreas Knopf, Ulrich Strassen

**Affiliations:** 1 Department of Otorhinolaryngology, University Clinic of Innsbruck, 6020 Innsbruck, Austria; 2 Department of Otorhinolaryngology, Technical University of Munich (TUM), 85354 Freising, Germany; 3 Institute of General and Surgical Pathology, TUM School of Medicine, Technical University of Munich (TUM), 81675 Munich, Germany; 4 Department of Nuclear Medicine, Technical University of Munich (TUM), 85354 Freising, Germany; 5 Institut für KI und Informatik in der Medizin, 81675 München, Germany; 6 Department of Dermatology and Venereology, Universitätsklinikum Hamburg-Eppendorf, Fleur Hiege Center for Skin Cancer Research, 20246 Hamburg, Germany; 7 Department of Otorhinolaryngology, Head and Neck Surgery, Albert-Ludwigs-Universität Freiburg, 79085 Freiburg, Germany

**Keywords:** pleomorphic adenoma, carcinoma ex pleomorphic adenoma, warthin tumor, salivary gland tumors, immunohistochemistry, SSTR2, tumor marker, theranostic, somatostatin receptor, radionuclide therapy

## Abstract

**Simple Summary:**

Currently, the diagnosis of salivary gland tumors using current imaging techniques is unreliable. In this study we examined salivary gland tumors and discovered that the pleomorphic adenoma, a tumor which should be surgically removed because it has a tendency to become malign, has a strong concentration of the somatostatin receptor 2. This characteristic may allow physicians to identify and potentially treat the tumor in a non-invasive manner.

**Abstract:**

Reliable preoperative diagnosis between salivary gland tumor entities is difficult. In this monocentric retrospective study, we examined the somatostatin receptor 2 (SSTR2) status of salivary gland tumors after salivary gland tumor resection via immunohistochemistry (IHC), and stains were compared in analogy to the HER2 mamma scale. A total of 42.3% of all pleomorphic adenoma (PA) tumors (42 of 99, 95% confidence interval 32.5–52.8%) demonstrated ≥20% of cells displaying the SSTR2 as compared to just 1% of all other tumors (1/160, 95% CI 0.02–3.4%). The other tumor was a neuroendocrine carcinoma. PA had a higher intensity of SSTR2 staining, with 90.9% staining ≥ an intensity of 2 (moderate). Tumors with an intensity of SSTR2 expression equal to or greater than 2 had an 89.9% likelihood of being a PA (95% CI: 82.2–95.0%, AUC: 0.928). Only one Warthin tumor demonstrated a ‘strong’ SSTR2 staining intensity. No Warthin tumor showed a percentage of cells staining for SSTR2 above ≥20%. This result demonstrates consistent and strong expression of SSTR2 in PAs as compared to Warthin tumors, which may allow physicians to utilize radioligand-somatostatin analog PET CT/MR imaging to diagnose the PA. SSTR2 positivity, if shown to be clinically relevant, may allow peptide receptor radionuclide therapy in the future.

## 1. Introduction

Salivary gland tumors account for 3–6% of all head and neck tumors [1]. The heterogeneity of this group is partially due to the diverse cell populations that are found in the salivary glands. The pleomorphic adenoma (PA) is one of the most common tumors of the salivary glands, accounting for 40–60% of all salivary gland tumors, with a growing incidence in the past 20 years [2]. The most common location is the parotid gland (85%), followed by the minor salivary glands (10%) and the submandibular gland. These benign tumors have a predilection for women between the ages of 30 and 60 [3].

Histologically, this tumor is described as a benign mixed tumor and is characterized by cellular pleomorphism with both epithelial and mesenchymal tissue. Pleomorphic adenomas have a low risk of malignant transformation into a Carcinoma ex pleomorphic adenoma (CXPA, 1–10%) and clinically present as slow-growing, painless tumors. Signs of malignancy, such as facial paralysis, are not typically observed. The risk for recurrence and malignancy increases with increasing tumor size, female gender, and young age and is proportional to the time the lesion is in situ [4].

Due to their persistent growth and risk for malignant transformation, the recommended course of therapy for the pleomorphic adenoma is total surgical excision without perforation of the pseudocapsule. If the pseudocapsule is perforated during surgical resection, there is a lifelong risk of recurrence. The risk of recurrence is, to some degree, dependent on histology. One can differentiate three subtypes of pleomorphic adenoma: the cellular, the classic, and the myxoid. Of these three types, the myxoid has the thinnest capsule and the highest likelihood of pseudopodia propagation [5]. To prevent recurrence, surgical excision is typically performed by partial or complete removal of the affected gland. Tumor operations of the parotid gland may, due to the location of the facial nerve, lead to facial muscle paralysis. Additionally, postoperative scarring after every operation increases the risk of a facial nerve lesion for repeat operations. In contrast, the Warthin tumor does not have a risk of malignant transformation and may be regularly observed as opposed to being surgically removed. A diagnostic tool that could discriminate between the two most common tumors would be valuable.

Commonly performed diagnostic examinations of tumors in the head and neck region include computerized tomography imaging, magnetic resonance imaging, and sonography. Superficial PA tumors located in the larger salivary glands are often well visualized using ultrasound. In ultrasound, they appear hypoechoic in character, demonstrate a smooth lobulated border, and may have a homogeneous or heterogeneous parenchyma with a posterior echo. Doppler sonography typically displays a poorly vascularized tumor. 

On computer tomography (CT) or magnetic resonance imaging (MRI), pleomorphic adenomas appear as well-circumscribed, lobulated tumors. In early development, tumors are often homogenous. As they increase in size, they often become more heterogeneous, and calcifications and partial necrosis may appear. In general, reflecting the abundant myxochondroid stroma, the pleomorphic adenoma has a hypodensity on CT and a very high signal on T2-weighted images, higher than the cerebrospinal fluid signal with higher apparent diffusion coefficient (ADC) values. Well-defined margins are present in most PAs, and PAs typically have a hypointense rim on T2-weighted images, indicating the fibrous capsule. An aggressive parotid tumor typically shows low signal intensity on T2-weighted images and an ill-defined tumor margin with an infiltrative border after contrast administration. Irregular margins and invasion into adjacent structures can indicate the presence of an infiltrating malignant parotid tumor [6]. MRI or CT are especially advantageous for tumors that affect the deeper parotid gland or minor salivary glands of the mucous tissues. Poorly circumscribed margins are the most accurate indicator of a malignant tumor; however, this characteristic is not seen in all malignant tumors, especially the smaller malignant tumors, which often resemble benign tumors when imaged [7]. While these diagnostic imaging techniques may generally give a good indication if a tumor is malignant or benign, an accurate and reliable diagnosis of a pleomorphic adenoma is difficult. Expert sonographers managed a sensitivity of 70% and a specificity of 95% in the diagnosis of pleomorphic adenoma via ultrasound [8]. The use of fine needle aspiration biopsy has been shown to improve the interpreted radiological results in discriminating between benign and malignant tumors with a sensitivity between 86% and 100% and a reported specificity between 90% and 100% [9,10]. The ability to accurately discriminate between benign tumors is worse, with a reported sensitivity of 72.4% [11]. A large meta-analysis of 5647 patients found that when considering both benign and malignant tumors, the sensitivity ranged between 72.4–88% and the specificity between 78.6–100% [12].

Imaging quality has steadily improved over the past two to three decades. However, sensitive and specific diagnostic imaging of head and neck tumor entities using imaging techniques is an ambitious goal that has largely been neglected in contemporary research. The first steps towards utilizing diagnostic tools for identifying said tumors include thoroughly understanding their histology.

Studies have investigated various common culprits associated with tumor growth. Fibroblast growth factor, platelet-derived growth factor (PDGF), Mucin 1 (MUC 1), p16, cyclin D1, and E2F proteins were observed to be more strongly expressed in recurrent PA as compared to non-recurrent PA. The pleomorphic adenoma gene 1 (PLAG1) is a zinc finger transcription factor and a proto-oncogene that may be visualized using immunohistochemistry and is strongly expressed in pleomorphic adenoma [13]. Evidence suggests that an oncogenic rearrangement of PLAG1, which is often found in PA as well as CXPA, is associated with tumorigenesis [14]. Other studies have investigated hormonal receptors such as progesterone and estrogen receptors or human epidermal growth factor receptor-2 (HER-2), a proto-oncogene associated with angiogenesis. These were, however, not shown to be strongly expressed in PA, recurrent PA, or CXPA [15]. It is unclear if PA arises from a single pluripotent cell or from more than one ‘stem cell’, though substantial evidence suggests it may well arise due to clonal expansion from a single pluripotent cell [14].

A recent case study described the incidental intensive tracer uptake of a pleomorphic adenoma in a patient who received a ^68^Ga-DOTATOC PET/CT [16]. An imaging technique that could sensitively and specifically identify the PA could be invaluable. However, further studies investigating this incidental tracer uptake have not been performed. The DOTATOC PET/CT utilizes a high concentration of somatostatin receptors in specific tumors to identify these. Somatostatin-receptor (SSTR) positron emission tomography (PET) CT, or PET MRI utilize radiotracers that bind to cells with the SSTR to visualize tumors expressing somatostatin receptors. The initial and primary intention for the design of radiolabeled SST imaging was to identify neuroendocrine carcinomas (NECs), a heterogenous group of tumors. However, researchers have found that SSTR imaging can also visualize inflammatory granulomas and autoimmune conditions.

One may distinguish between five different SSTR subtypes (1, 2A/B, 3, 4, and 5), which are expressed in varying degrees in diverse neoplastic tumors and tissues [17]. In addition to its well-known anti-secretory, anti-nociceptive, and anti-inflammatory functions, researchers have discovered that somatostatin and its receptors also inhibit cell growth and angiogenesis [18]. Somatostatin receptors have been identified in the metaphysis next to hypertrophic cartilage [19]. Cells that stain for somatostatin also stain for alkaline phosphatase, which suggests that they may play a role in precursor cells to osteoblasts, which play a role in embryonic bone formation, including enchondral ossification [20]. The variable cytomorphology of pleomorphic adenoma regularly includes cartilage and chondromyxoidal tumors [21]. Besides the formation of myxoid-rich parenchyma, bone formation has also been described in pleomorphic adenomas [22].

Immunohistochemistry (IHC) is the routine standard method utilized to determine the distribution and prevalence of SSTR subtypes in certain tissues. It has an established value and reliability that have been widely supported in different types of tumors. Immunohistochemistry analysis of tumors has been demonstrated as a sensitive and specific method for confirming receptors in cells [23,24]. The goal of this study is to retrospectively examine tumors of the salivary glands that were removed during salivary gland operations using immunohistochemistry analysis to determine if the SSTR2 receptor is expressed as a differentiation in the SSTR2 status between the two most common tumors, the Warthin tumor and the PA, which may allow a diagnostic avenue to differentiate them.

## 2. Materials and Methods

The study protocol is in accordance with the Declaration of Helsinki. The Institutional ethics board of the Medical Faculty, Technical University of Munich, reviewed and approved the protocol (Ethics committee file number: 2022-591-S-KH).

In this monocentric retrospective study, the electronic patient medical database of the Technical University of Munich School of Medicine, Klinikum rechts der Isar, was searched from March 2016 to December 2019 for patients who underwent partial or total extirpation of the parotid gland. Patients with cysts were removed from the collective as these can be sonographically well differentiated from other parenchymatous tumors. Deidentified data points, including histology, were entered into an Excel (V16.19) spreadsheet.

For immunohistochemistry (IHC), 3 µm thick sections of formalin-fixed and paraffin-embedded (FFPE) tissue blocks were prepared. Subsequently, sections were incubated with the SSTR2A antibody (ZYTOMED Systems, Berlin, Germany; 1:100) for 32 min. A Ventana BenchMark Ultra automated stainer with the iView DAB Kit was utilized for immunohistochemical stainings (Ventana Medical Systems, Oro Valley, AZ, USA). Finally, tissue sections were counterstained with hematoxylin. Positive controls were prepared to serve as quality assurance.

**Figure 1:**
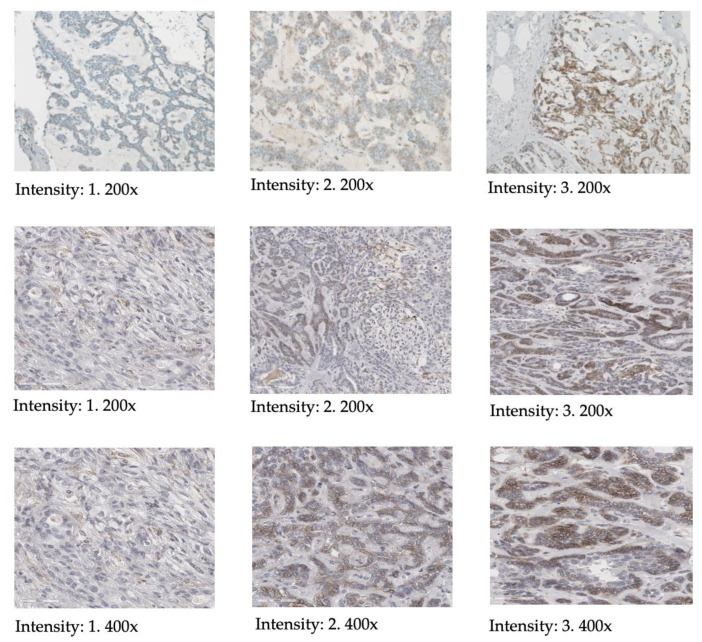
Illustration of scoring of the SSTR2 intensity according to the HER2 scoring system, including magnification.

IHC scoring was performed using light microscopy and the HER2 scoring system typically applied for breast cancer hormone receptor analysis (see Table 1 and Figure 1) [25]. Membranous staining was scored as specific and evaluated as previously described for pancreatic neoplasms [26]. In short, score 0 was assigned for cases without membranous staining in <10% of cells; score 1 for mild membranous staining in >10% of cells; score 2 for moderate membranous staining in >10% of cells; and score 3 for strong membranous staining in >10% of cells. The percentage of cells in the tumor (0–100%) expressing SSTR2A as well as the intensity (0 = none, 1 = mild, 2 = moderate, 3 = strong) were noted for each tumor.

Statistical analysis of the data was performed using R 4.2.1 (R Foundation for Statistical Computing, Vienna, Austria). The percentage of cells expressing SSTR2 was categorized into classes “0–19%”, “20–39%”, “40–59%”, “60–79%” and “80–100%”. Absolute and relative frequencies of the observed percentage of cells expressing SSTR2 and intensity of SSTR2 expression were determined and stratified for tumor diagnosis (detailed diagnosis and PA versus non-PA). For relevant proportions, exact 95% confidence intervals (Clopper-Pearson intervals) were estimated and presented. A receiver-operating characteristic (ROC) analysis was performed to evaluate the association between positivity or intensity and the tumor entity (PA versus non-PA).

A classification tree was fitted to the data to determine which data combination could be used to discriminate between Warthin tumors and PA. Additionally, logistic regression models with signal intensity and percentage of SSTR2-expressing cells as single predictor variables were fitted to the data and compared to a multivariable model including both covariates as independent variables. The models were compared using likelihood tests.

## 3. Results

A total of 354 patients were identified in the clinical database. 30 non-PA tumors were excluded because the respective histological samples were not present in the pathology department and could not be examined. Furthermore, 95 pathological samples were excluded, as they represented general parenchymatous changes due to autoimmune disorders or because they were non-glandular tumors or cysts. Of the remaining 259 patients, 99 were PA, 129 were Warthin tumors, and 31 had other tumors. A detailed summary of all tumors, including the percentage of cells expressing SSTR2 examined using IHC, is seen in Table 2. Table 3 described the percentage of total cells expressing the SSTR2 stratified according to PA and all other tumors. 

Immunohistochemical analysis of these 259 tumors (99 PA and 160 non-PA) revealed that the total percentage of cells that were positively stained for SSTR2 was higher in PA as compared to other tumors. A total of 42 tumors had an SSTR2 cell count equal to or above 20%. Of these, only one was not a PA tumor. 42.3% of all PA tumors (42 of 99, 95% confidence interval 32.5–52.8%) demonstrated ≥20% of cells displaying the SSTR2 as compared to just 1% of all other tumors (1/160, 95% CI 0.02–3.4%).

Only one other tumor, a neuroendocrine carcinoma, was observed to demonstrate more than 20% of cells staining for SSTR2. All other tumors that had a level equal to or above 20% were PA.

**Figure 2:**
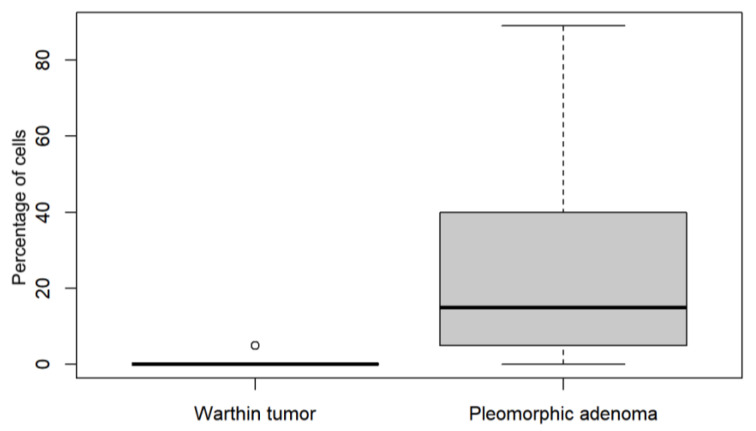
Comparison of the percentage of total SSTR2 cell expression between Warthin tumors and pleomorphic adenoma.

A representation of the total SSTR2 cell expression in Warthin tumors versus PA as seen in IHC is illustrated in Figure 2. In comparison, pleomorphic adenoma tumors demonstrated a higher expression of SSTR2.

Furthermore, PA were found to have a high intensity of SSTR2 staining, with 90.9% (95% CI 83.4–95.8%) staining ≥ an intensity of 2 (moderate). A grade of ‘one’ (mild) was given to only two non-PA tumors: the adenoid cystic carcinoma and the basal cell adenoma. When further considering only the non-PA tumors, a score of ‘two’ (moderate) was given to a MALT, a mucoepidermoid carcinoma, and a primary squamous cell carcinoma. A score of ‘three’ (strong) was given to three basal cell adenoma tumors: a CXPA, a NEC, a case of primary squamous cell cancer, and one Warthin tumor. A tabularized summary of this data may be seen in Table 4.

Of the pleomorphic adenomas, 9 received a score of 0, with 8 receiving a score of ‘two’ and 82 receiving a score of ‘three’. Notably, the CXPA received an intensity rating of ‘three’.

**Figure 3:**
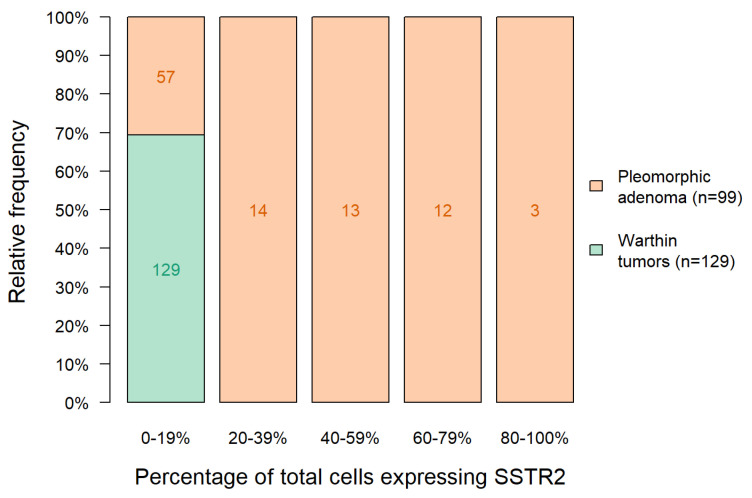
Relative frequency of SSTR2 intensity with tumor stratification according to Warthin tumor vs. PA.

The relative frequency of SSTR2 intensity with tumor stratification according to PA vs. Warthin tumors is shown in Figure 3.

**Figure 4:**
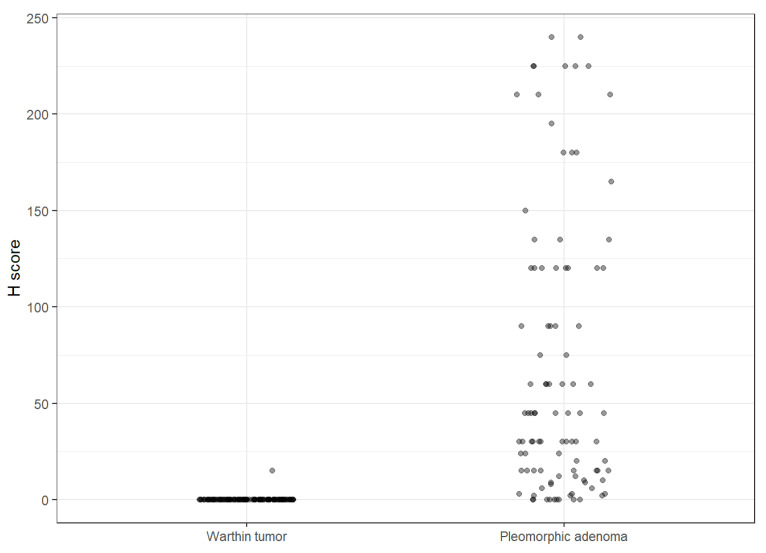
Illustration of the H-score analysis of Warthin tumor versus PA.

In order to analyze the intensity of SSTR2 uptake, a modified histoscore (H-score) ROC was utilized. The H-score was calculated by multiplying a semi-quantitative assessment of both the intensity of staining and the percentage of positive cells. An illustration of the H-score analysis of Warthin tumor versus PA is seen in Figure 4. 

Different statistical methods were applied in order to investigate whether a combination of the information on the percentage of SSTR2-expressing cells and the signal intensity could be used to increase the discrimination between pleomorphic adenoma and Warthin tumors.

A classification tree was fitted to the data using the function tree provided in the R package partykit [27,28]. In the tree procedure, intensity with a cutoff value of “0” vs. “1–3” was selected as the only split. Additionally, logistic regression models with signal intensity and percentage of SSTR2-expressing cells as single predictor variables were fitted to the data and compared to a multivariable model including both covariates as independent variables. A comparison of the models using likelihood ratio tests showed a significant improvement in the model fit when the model with both predictor variables was compared to the model using percentage of SSTR2 expressing cells only (χ^2^ = 18.6, *p* < 0.001), but not when the combination model was compared to the model using intensity only (χ^2^ = 1.904, *p* = 0.168).

This indicates that in our data, the discriminatory ability—which is already very good using signal intensity alone (area under the ROC curve of 0.95)—could not be significantly improved by combining information on intensity and percentage of SSTR2-expressing cells.

**Figure 5:**
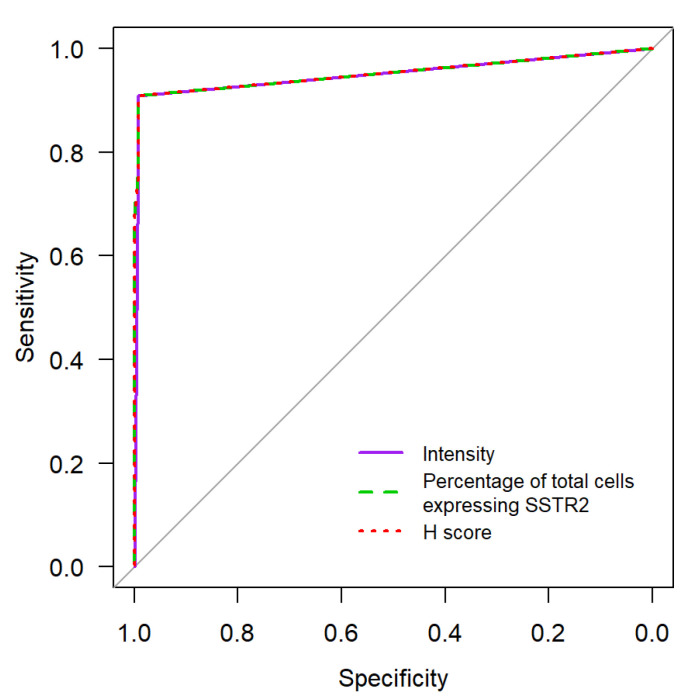
ROC-Curve Comparison of “Intensity of SSTR2 expression” vs. “Percentage of totals cells expressing SSTR2” and H-score towards predicting the presence of PA.

The “intensity of SSTR2 expression” alone was shown to be a strong predictor of the presence of PA. This may be seen in the ROC (Figure 5, AUC: 0.928, 95% CI 0.894–0.962), with an optimal cut-off value of 2. Tumors with an intensity of SSTR2 expression equal to or greater than 2 had an 89.9% likelihood of being a PA (95% CI 82.2–95.0%).

## 4. Discussion

Of the 259 tumors examined, 129 were Warthin tumors, 99 were PA, and 31 were other tumors. A higher prevalence of Warthin tumors as compared to PA has been described in Germany [29,30,31]. All Warthin tumors examined in this study showed an SSTR2 cell count below 20%. Of all the non-pleomorphic tumors (n = 160), only the NEC was shown to have an SSTR2 cell count equal to or higher than 20%. Of the 42 tumors with ≥20% SSTR2 cell expression, 41 were PA. Figure 2 illustrates the increased expression of SSTR2 in PA cells in comparison to Warthin tumors. 42.3% of all PA tumors (42 of 99, 95% confidence interval 32.5–52.8%) demonstrated ≥20% of cells displaying the SSTR2 as compared to just 1.0% of all other tumors (2/207, 95% CI 0.1–3.4%).

The PA were found to exhibit an increased intensity of SSTR2 staining, with 90.9% (95% CI 83.4–95.8%) staining ≥ an intensity of 2 (moderate). Some individual tumors demonstrated strong (grade 3) SSTR2 intensity. These include the basal cell adenoma (n = 3), a case of primary squamous cell cancer (n = 1), and one Warthin tumor (n = 1). While these individual samples demonstrated a strong intensity, none of them were found to have a high percentage of total cells that express SSTR2. Unsurprising was the strong SSTR2 intensity in both the neuroendocrine carcinoma (NEC) and CXPA tumors. The CXPA demonstrated an SSTR2 cell count between 0 and 19% and was shown to have a strong intensity (grade of 3). Supported by a high number of individual cases of PA (n = 99) and Warthin tumors (n = 129), the higher “percentage of cells staining for the SSTR2” and “intensity of SSTR2 staining” in PA is clear.

While PAs have a risk of malignant transformation, Warthin’s tumors do not. Consequently, PA should be surgically removed, while Warthin tumors may be monitored. A diagnostic tool that could differentiate the two entities would therefore be valuable.

A potential limitation of our findings, however, is the described low SSTR2 visualization in a subpopulation of PA. In a total of eight PA, zero SSTR2 expression could be visualized in IHC. In some cases of PA and a case of CXPA, the total percentage of cells was low, but the intensity of SSTR2 was high. This means that while SSTR positivity in DOTATOC is a strong indicator for pleomorphic adenoma, SSTR negativity does not allow for any conclusion on a specific tumor entity or the risk of malignancy.

Gene expression analysis or other confirmatory assays should be performed in future studies to corroborate the results of this study.

The case study performed by Laurens et al. [16] demonstrated a potentially unknown characteristic of the pleomorphic adenoma: that it can be identified using the DOTATOC PET/CT. This characteristic may be due to the presence of the somatostatin receptor in PA. The presence of SSTR2 in PA may be embryologically explained by their pleomorphic histology, which includes mesenchymal tissue, which has been shown to display SSTR2 [32]. The immunohistochemistry analysis of parotid gland tumors identified the strong presence of the SSTR2 receptor in PA. This feature has previously not been described in the literature. The only other tumor with a comparably high cell expression of SSTR2 and high intensity grading was a NEC. The data presented demonstrates a strong association between “Intensity of SSTR2 expression” and the presence of pleomorphic adenoma (95% CI 82.2–95.0%). When excluding the CXPA and NEC tumors from the “non-PA” group, this statistic improves.

Preoperative diagnostic imaging plays an important role in surgery, allowing surgeons to prepare for an operation. Before an operation, an ultrasound evaluation should be performed. Sonography is a quick, non-expensive, and non-invasive imaging technique that helps visualize superficial tumors. Tumors in the deep parotid lobe, however, are more difficult to image using sonography and may require a CT or MRI, which permit a better view of deeper tissue.

Tumors may be benign or malignant and have varying degrees of malignancy as well as proliferation, which require different surgical approaches. Contemporary preoperative diagnostic imaging of salivary gland tumors can rarely specifically identify tumors, preventing surgeons from operating with certainty. Differential diagnoses include benign tumors such as the PA, the Warthin tumor, lymphangiomas, cysts, and granulomatous-associated tumors. However, approximately 20% of parotid tumors are malignant, with a malignancy rate of up to 80% in minor saliva glands, including, for example, lymph node metastases, acinic cell carcinoma, adenoid cystic carcinoma, mucoepidermoid carcinoma, and lymphomas [33]. Recently, artificial intelligence using radiomic analysis was reported for discriminating benign and malignant salivary gland tumors based on conventional anatomical MRI features [34]. However, the clinical relevance of this method has not been validated. While PA usually presents as well-circumscribed, rounded hypoechoic homogenous tumors in ultrasound imaging, highly aggressive malignant tumors such as NEC and CXPA present as irregular masses with non-homogenous parenchyma and are therefore easily distinguishable from PA [35,36].

Certain tissues and tumors, including neuroendocrine tumors, express receptors that bind to hormones, including somatostatin. SSTR overexpression in certain tumors combined with somatostatin analogues (SSAs) allows both specific imaging of said tumor as well as a non-invasive targeted tumor therapy. SSAs may be labeled with radionuclides, which undergo radioactive decay. Depending on which radionuclide is ligated, the tumor may be diagnostically located (X-ray emission through gamma-ray photon production) or therapeutically targeted with the emission of alpha/beta particles [37]. Commonly used SSAs include “DOTA-peptides”, DOTATOC (DOTA-Tyr3-octreotide), and DOTATATE (DOTA-Tyr3-octreotate), which may be ligated to a radiopharmaceutical such as gallium-68 (^68^Ga), which produces positrons (positron emission tomography, PET). DOTATOC or DOTATATE PET/CT or MRI imaging analysis is a diagnostic imaging tool typically utilized for the detection of neuroendocrine tumors. Visualization of specific tumor cells that express SSTR is achieved by attaching a radioactive isotope (^68^Gallium) with quick radioactive decay to a ligand (the stable somatostatin analogue: DOTATOC or DOTATATE) that binds to SSTR.

A large meta-analysis of ^68^Ga-DOTA-conjugated PET/CTs suggests basal physiological tracer uptake in healthy salivary glands. As mentioned in the referenced study, PET/CTs are increasingly performed, and as such, rates of incidental findings will also increase, and research in this field will help clinicians deal with unanticipated findings [38]. In the case study presented by Laurens et al., a strong and distinctive uptake is noticeable in the pleomorphic adenoma, while the otherwise healthy salivary gland tissue has no uptake. A basal physiological SSTR2 presence has been reported via IHC in healthy salivary tissue. However, physiological organ tracer-uptake in DOTATOC PET/CTs does not necessarily hinder discriminating tumors with high SSTR2 concentrations in said organs, as tracer-uptake values in physiological tissue vary from those of tumors that strongly express SSTR2 [39,40]. This issue should, however, be further examined through clinical examination of DOTATOC PET/CTs.

Tracer-uptake in DOTATOC PET/CTs has also been reported in other tumors, such as lymphoepithelial carcinoma, a rare tumor entity with only a few hundred reported cases worldwide [41]. A study found a high concentration and intensity of SSTR2 when examining nine Epstein–Barr virus-positive nasopharyngeal carcinomas using immunohistochemistry [42]. An examination of a collective of malignant salivary gland tumors using immunohistochemistry indicates that some tumors, such as the mucoepidermoid carcinoma, also exhibit SSTR2 [43]. In this study, of two cases of mucoepidermoid carcinoma, one had moderate SSTR2 expression and one had no SSTR2 expression.

Targeted radionuclide therapy (TRT) has been successfully employed for over 75 years, since the first radioiodine thyroid treatment. TRT is used to treat adenomas in anatomically sensitive locations (thyroid or pituitary adenomas) as well as malignant cancers. The strong expression of the somatostatin receptors in pleomorphic adenomas in our study may be a hint that a non-invasive peptide receptor radionuclide therapy (PRRT) might be feasible for PA in the future. Such a treatment could, for example, be considered as an alternative to re-operation in patients who have been operated on multiple times in the parotid region due to metastatic PA recurrence. Repeated operations in the parotid gland area are associated with an increased risk of facial nerve injury due to scarring [44]. A further indication could be for carcinoma ex pleomorphic adenoma (CXPA) or as a neoadjuvant treatment for large, difficult-to-operate tumors.

Studies suggest that SSTR2 may be a strong mediator of tumor growth. Neuroendocrine carcinomas (NECs) displaying SSTR2 have been found to have a more favorable prognosis and a better response to treatment using SSAs [45,46]. This speaks favorably for a PRRT for PAs or CXPAs. In the NETTER-1 phase 3 study investigating the use of lutetium-177-DOTATATE in the treatment of patients with advanced metastasized midgut neuroendocrine tumors, it was demonstrated that ^177^Lu-DOTATATE significantly improved overall progression-free survival [47].

A further line of treatment may be the direct pharmaceutical use of synthetic somatostatin analogues, which have been shown to reduce cell proliferation in tumors expressing SSTR2. Somatostatin is a hormone that is typically characterized as an endogenous neuropeptide well known as an inhibitor of growth hormone. The native somatostatin peptide only has a short plasma half-life of 1–3 min, which limits its therapeutic utility. However, somatostatin analogues have been created that are more stable, have longer half-lives, and have varying affinities for the different somatostatin receptors [48]. Somatostatin analogues were initially conceived to reduce carcinoid syndrome resulting from NEC-related hormone hypersecretion. However, recent studies have demonstrated in prospective placebo-controlled and randomized studies that SSAs (octreotide and lantreotide) also reduce tumor progression in NECs displaying SSTR2 [49,50]. If the strong presence of SSTR2 in PA is shown to have a clinical meaning, an SSA treatment could potentially be used to prevent tumor progression in patients with serious comorbidities that pose a high risk for general anesthesia and/or surgery or in cases where a conservative, non-invasive therapy is preferable.

## 5. Conclusions

In this monocentric retrospective study, a large collective sample of 259 tumors of the parotid gland was examined using immunohistochemistry. This sample is the largest described immunohistochemistry examination of SSTR2 in salivary gland tumors. This study demonstrates the increased presence and expression of the somatostatin receptor 2 in pleomorphic adenomas in comparison to the Warthin tumor, thereby explaining the strong tracer-uptake in the DOTATOC PET/CT in a pleomorphic adenoma described in the case study by Laurens et al. tumors with an intensity of SSTR2 expression greater than or equal to 2 were strongly associated with the presence of a pleomorphic adenoma (89.9%, 95% CI 82.2–95.0%). Furthermore, the SSTR2 may allow a reliable distinction between the two most common salivary gland tumors, the Warthin tumor and the PA. Being able to differentiate between the Warthin tumor, which does not have a risk for malignant transformation, and the PA, which has a risk of malignant transformation and should be surgically removed, would be a useful diagnostic tool and may prevent unnecessary operations.

Although this study in its scope cannot confirm the clinical value of SSTR2 in PA, the strong expression of SSTR2 may allow researchers to accurately and selectively visualize PAs using DOTATOC or DOTATATE PET/CT or PET/MRI imaging. The newly described presence and overexpression of SSTR2 could open an avenue for diagnostic imaging and therapy. This feature may have a useful clinical impact and could allow physicians to accurately diagnose and potentially treat pleomorphic adenoma or carcinoma ex pleomorphic adenoma via somatostatin analogues or a non-invasive peptide receptor radionuclide therapy. The results of this retrospective analysis should be corroborated in a prospective study setting.

## Figures and Tables

**Table 1:** IHC Analysis using a HER2 scoring system, modified according to Tripathy, Mishra et al., 2018.

Staining	Score	Evaluation
No staining was observed; faint membrane staining was observed in ≤10% of tumor cells	0	None
Incomplete, barely visible staining in >10% of tumor cells	1	Mild
Incomplete and/or weak circumferential staining in >10% of tumor cells, or complete, intense staining in ≤10% of tumor cells	2	Moderate
Complete, intense staining in >10% of tumor cells	3	Strong

**Table 2:** Percentage of cells expressing SSTR2.

Percentage of Cells Expressing SSTR2	0–19%	20–39%	40–59%	60–79%	80–100%	Total
Warthin tumor	129	0	0	0	0	129
Pleomorphic adenoma (PA)	57	14	13	12	3	99
Basal cell adenoma	9	0	0	0	0	9
Oncocytoma	7	0	0	0	0	7
Primary squamous cell cancer	3	0	0	0	0	3
MALT	2	0	0	0	0	2
Mucoepidermoid carcinoma	2	0	0	0	0	2
Myoepithelioma	2	0	0	0	0	2
Secretory carcinoma	1	0	0	0	0	1
Neuroendocrine Carcinoma	0	0	0	0	1	1
Lymphoma	1	0	0	0	0	1
Carcinoma ex pleomorphic adenoma (CXPA)	1	0	0	0	0	1
Adenoid cystic carcinoma	1	0	0	0	0	1
Reticulary myoepithelioma	1	0	0	0	0	1
Total	216	14	13	12	4	259

**Table 3:** Percentage of total cells expressing SSTR2 stratified according to PA and non-PA.

Percentage of Total Cells Expressing SSTR2		
Positivity in %	All Other Tumors (n = 160)	Pleomorphic Adenoma (n = 99)
0–20	159	57
20–40	0	14
40–60	0	13
60–80	0	12
80–100	1 *	3

* Neuroendocrine carcinoma.

**Table 4:** Intensity of SSTR2 expression.

Intensity of SSTR2 Expression (Grading: 0–3) *					
Grading	None (0)	Mild (1)	Moderate (2)	Strong (3)	Total
Warthin tumor (Cystadenolymphoma)	128	0	0	1	129
Pleomorphic adenoma (PA)	9	0	8	82	99
Basal cell adenoma	5	1	0	3	9
Oncocytoma	7	0	0	0	7
Primary squamous cell cancer	1	0	1	1	3
MALT	1	0	1	0	2
Mucoepidermoid carcinoma	1	0	1	0	2
Myoepithelioma	2	0	0	0	2
Secretory carcinoma	1	0	0	0	1
Neuroendocrine Carcinoma	0	0	0	1	1
Lymphoma	1	0	0	0	1
Carcinoma ex pleomorphic adenoma (CXPA)	0	0	0	1	1
Adenoid cystic carcinoma	0	1	0	0	1
Reticulary myoepithelioma	1	0	0	0	1

* According to the HER2-Mama scale.

## Data Availability

The data presented in this study are available in this article.
